# The Influence of Physical Activity and Epigenomics On Cognitive Function and Brain Health in Breast Cancer

**DOI:** 10.3389/fnagi.2020.00123

**Published:** 2020-05-08

**Authors:** Monica A. Wagner, Kirk I. Erickson, Catherine M. Bender, Yvette P. Conley

**Affiliations:** ^1^School of Nursing, University of Pittsburgh, Pittsburgh, PA, United States; ^2^Department of Psychology, University of Pittsburgh, Pittsburgh, PA, United States; ^3^Discipline of Exercise Science, College of Science, Health, Engineering and Education, Murdoch University, Perth Campus, Murdoch, WA, Australia; ^4^Department of Human Genetics, University of Pittsburgh, Pittsburgh, PA, United States

**Keywords:** epigenomics, physical activity, cognitive function, breast cancer, DNA methylation, brain health

## Abstract

The risk of breast cancer increases with age, with the majority of women diagnosed with breast cancer being postmenopausal. It has been estimated that 25–75% of women with breast cancer experience changes in cognitive function (CF) related to disease and treatment, which compromises psychological well-being, decision making, ability to perform daily activities, and adherence to cancer therapy. Unfortunately, the mechanisms that underlie neurocognitive changes in women with breast cancer remain poorly understood, which in turn limits the development of effective treatments and prevention strategies. Exercise has great potential as a non-pharmaceutical intervention to mitigate the decline in CF in women with breast cancer. Evidence suggests that DNA methylation, an epigenetic mechanism for gene regulation, impacts CF and brain health (BH), that exercise influences DNA methylation, and that exercise impacts CF and BH. Although investigating DNA methylation has the potential to uncover the biologic foundations for understanding neurocognitive changes within the context of breast cancer and its treatment as well as the ability to understand how exercise mitigates these changes, there is a dearth of research on this topic. The purpose of this review article is to compile the research in these areas and to recommend potential areas of opportunity for investigation.

## Introduction

Despite tremendous research efforts, breast cancer continues to be the second leading cause of all cancer deaths worldwide and the most commonly diagnosed cancer among women (Bray et al., 2018). In the United States, the lifetime probability of being diagnosed with cancer is 38.4% (National Cancer Institute, 2018). Currently, more than 3.1 million women are living with breast cancer in the United States (Siegel et al., 2019). Aging is a primary risk factor for cancer due to the gradual decline in physiological integrity experienced with aging that decreases the integrity of the cell and leaves it vulnerable to disease, such as cancer. A phenotype of accelerated aging has been associated with breast cancer and breast cancer treatment (López-Otín et al., 2013; Aunan et al., 2016). Advances in science and technology have led to earlier cancer detection and treatments that have resulted in better overall and disease-free survival rates. In 1976, the 5-year survival rate for women with breast cancer was 75%, but that number has risen to 90% in 2019 (Chang et al., 2019; Siegel et al., 2019). As the number of individuals surviving cancer continues to grow, so does the number of those who are living with the side effects of their cancer and cancer treatment. For this reason, there is an increasing demand for research devoted to the prevention or amelioration of unwanted late and long-term effects of cancer and its treatment.

Breast cancer and its treatment can produce significant decreases in neurocognitive function in 25–75% of women with the disease (Wefel et al., 2004a; Bender et al., 2014, 2018). Between 30% and 35% of women with breast cancer have poorer cognitive function (CF; compared to healthy age and education-matched women) before they begin adjuvant therapy (Wefel et al., 2004b; Hardy et al., 2018). This suggests that factors in addition to cancer therapy contribute to poorer CF in this group. These neurocognitive changes compromise psychological well-being, decision making, performance of daily activities, employment, and adherence to cancer therapy (Bender et al., 2014, 2015). Unfortunately, little is known about the mechanisms that underlie the neurocognitive changes in women with breast cancer and its therapy, which in turn limits the development of effective treatment and prevention strategies (Falk and Dickenson, 2014; Borrie and Kim, 2017; Fukuda et al., 2017; Boyette-Davis et al., 2018). In contrast, exercise has been studied as a promising approach to positively impact CF and reduce the risk of cognitive loss and impairment (Erickson et al., 2019). However, we still have a poor understanding of the mechanisms by which exercise influences brain health in humans. We consider here the role of DNA methylation: (a) there is evidence suggesting that DNA methylation, an epigenetic mechanism for gene regulation, impacts CF and overall BH in the general population (Masser et al., 2017; Liu et al., 2018; Marioni et al., 2018; Gaiteri et al., 2019); and (b) there is evidence that exercise influences both DNA methylation and CF (Marioni et al., 2015; Fernandes et al., 2017; McCullough et al., 2017; Gale et al., 2018; McEwen et al., 2018; Voisey et al., 2019). Thus, changes in DNA methylation may reflect one mechanism by which exercise enhances cognitive and BH while also mediating the BH changes related to breast cancer. The purpose of this review is to summarize the research in these areas, provide a thoughtful and critical review of the field indicating that DNA methylation might be an important mechanism of exercise-induced improvements in BH, and recommend potential areas of opportunity for future investigation.

## Effect of Breast Cancer and Treatment on Brain Aging

**Figure 1:**
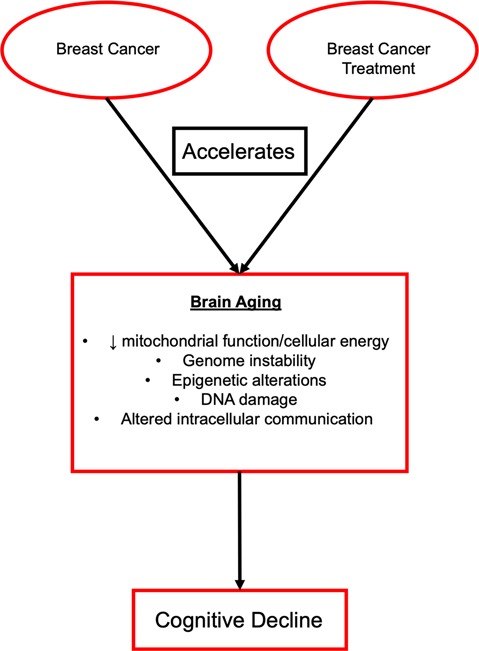
Effects of breast cancer and breast cancer treatment on brain aging.

Cellular aging includes changes to a variety of processes including the attrition of telomeres, decline in mitochondrial function and cellular energies, genome instability, epigenetic alterations, DNA damage that affects the suppressor checkpoints and other markers of cellular senescence, and altered intracellular communication (Table 1, Aunan et al., 2016; Chang et al., 2019). These hallmarks can be grouped into categories such as damage to cellular function (telomere attrition, genome instability, and epigenetic alterations), responses to the damage in cellular function (a decline of mitochondrial function and cellular energies, DNA damage that affects cell suppressor checkpoints and other markers of cellular senescence), and foundations of the clinical phenotype (altered intracellular communication; Aunan et al., 2016). These characteristics provide a basis for the complex biological connections between aging and cancer (Figure 1). The complexities of altered biological function with aging are also the hallmarks of cancer growth and include the ability of the cell to sustain rapid signaling, elude growth suppressors, stimulate invasion and metastasis, enable the immortality of replication, produce angiogenesis, and evade death (Hanahan and Weinberg, 2011). Each of these hallmarks is unique in function, but they all work together to support the growth of tumors and metastasis. New treatments are generally designed to work against these functions to stop tumor growth and the spread of disease. For example, epidermal growth factor receptor (EGFR) inhibitors are a type of targeted therapy that is designed to specifically target and block EGFR to halt the growth of cancer cells by blocking the EGFR protein, which plays a prominent role in tumor growth. Recent reviews on aging (López-Otín et al., 2013; Aunan et al., 2016), cancer (Hanahan and Weinberg, 2011), as well as the effect of breast cancer treatment on cellular aging (Chang et al., 2019) have covered the molecular mechanisms of these topics in greater depth.

**Table 1:** Exemplar hallmarks of molecular aging.

Hallmark	Description
Attrition of telomeres	Most mammalian somatic cells do not express telomerase, an enzyme that is responsible for replicating the terminal ends of linear DNA molecules. Therefore, the DNA sequences at the end of the chromosome progressively lose their telomere protection with each new cell division (López-Otín et al., 2013; Chang et al., 2019).
A decline in mitochondrial function and cellular energies	Declines in mitochondrial function and mutations in mitochondrial DNA appear to affect cellular energetics. Elevated levels of ROS resulting from mitochondrial dysfunction may decrease apoptosis and lead to resistance of chemotherapeutic agents thereby promoting breast cancer malignancy (López-Otín et al., 2013; Chang et al., 2019)
Genome Instability	Over time DNA damage accumulates in normal cells as the result of endogenous cellular activity such as DNA replication errors or DNA damage due to ROS (López-Otín et al., 2013; Chang et al., 2019). These damages not only lead to accelerated aging, but also make the cell vulnerable to cancer development.
Epigenetic Alterations	Epigenetic changes are alterations in gene expression that do affect the DNA sequence. These changes involve processes such as posttranslational histone modifications, DNA methylation patterns, and chromatin remodeling. Aging cells experience random DNA methylation drift creating mosaic aging stem cells that could lead to cancer (López-Otín et al., 2013; Aunan et al., 2016; Chang et al., 2019).
DNA damage that affects cellular senescence	Cellular senescence is by which a cell ceases to divide. The primary objective of senescence is to inhibit the proliferation of impaired cells and to mark the cells for destruction by the immune system. The process is associated with aging and age-related conditions. In older individuals, the widespread damage and poor clearance of senescence results in cell accumulation, which contributes to aging (López-Otín et al., 2013; Aunan et al., 2016; Chang et al., 2019).
Altered intracellular communication	As part of aging, inflammatory reactions increase leading to alterations in neurohormonal signaling. There is also a decrease in immunosurveillance against premalignant cells and pathogens and a change in the structure of both the extracellular and pericellular environments (López-Otín et al., 2013).

Estrogen loss is one mechanism through which aging may be accelerated resulting in cognitive decline in women with breast cancer. Over three-quarters of women with breast cancer in the United States are postmenopausal at diagnosis (DeSantis et al., 2016), 96% percent of these women are diagnosed with hormone receptor-positive disease (Clark et al., 1984; Osborne, 1998; Cheang et al., 2015), and the majority of these women will receive aromatase inhibitor therapy (Bender et al., 2014) that dramatically reduces estrogen. Estrogen exposure augments memory and learning and influences areas of the brain, such as the hippocampus, that are both rich in estrogen receptors and support episodic memory function (Bean et al., 2014; Duarte-Guterman et al., 2015; Hadjimarkou and Vasudevan, 2018; Korol and Wang, 2018; Paletta et al., 2018). Decreasing levels of estrogen are associated with cognitive decline (Luine, 2014; Frick, 2015; Gholizadeh et al., 2018; Yoon et al., 2018). Treatment for breast cancer often further reduces estrogen levels. Women who are on aromatase inhibitor therapy to reduce breast cancer occurrence can experience up to a 98% inhibition of the aromatase enzyme that leads to reduced estrogen (Brueggemeier et al., 2005; Kang et al., 2018) while women who receive chemotherapy also experience estrogen deprivation that has been associated with osteoporosis (Jonat et al., 2002; Ottanelli, 2015).

Estrogens serve a neuroprotective role against neurodegeneration. A recent review describes the neuroprotective effect of estrogen and the suggested mechanisms by which estrogen achieves this neuroprotection (Siddiqui et al., 2016). Estrogens have been cited to increase the expression of genes important for cell survival; shield neurons against injury due to oxidative stress, lack of glucose, and certain toxicities (glutamate, amyloid beta-peptide, iron sulfate); and lower the risk of cognitive decline and neurological deficits in women (Siddiqui et al., 2016). Estrogens exert neuroprotective properties *via* direct and indirect gene regulation mechanisms (Klinge, 2009). Direct gene activation is accomplished through nuclear binding estrogen receptors (alpha and beta) which serve as ligand-activated transcription factors. Indirect activation is the result of estrogen activation of plasma-associated estrogen receptors, which initiates an intracellular signaling cascade that results in altered gene expression (Klinge, 2009).

There are also epigenetic changes that occur with breast cancer and its treatment that can influence brain aging. Evidence suggests that abnormal DNA methylation patterns are well-established features of cancer and aging (Singhal et al., 2016; Pérez et al., 2018). Age is recognized as an important risk factor for cancer, but the DNA methylation patterns that serve as a link between aging and cancer are complicated and not well understood (Pérez et al., 2018). Some DNA methylation patterns in normal breast tissue are associated with heightened breast cancer risk (Daraei et al., 2017; Johnson et al., 2017; Hofstatter et al., 2018). The estrogen receptor 1 gene promoter is highly methylated in women with increased age indicating a possible mechanism by which breast cancer tissue is at a greater risk for developing cancer (Daraei et al., 2017). Environmental exposures such as alcohol intake and smoking disrupt the placement of methyl groups on the epigenome, leading to an increased risk for the development of breast cancer particularly in regulatory regions of DNA, including MYC proto-oncogene and CCTC-binding factor, that are further aggravated in cancer (Johnson et al., 2017). It has also been shown that women with breast cancer display significant acceleration of epigenetic age (an estimate of biological age based on DNA methylation patterns) in normal nearby breast tissue when compared to samples from unaffected women (Hofstatter et al., 2018).

## Effect of Breast Cancer and Treatment on Cognitive Function

**Figure 2:**
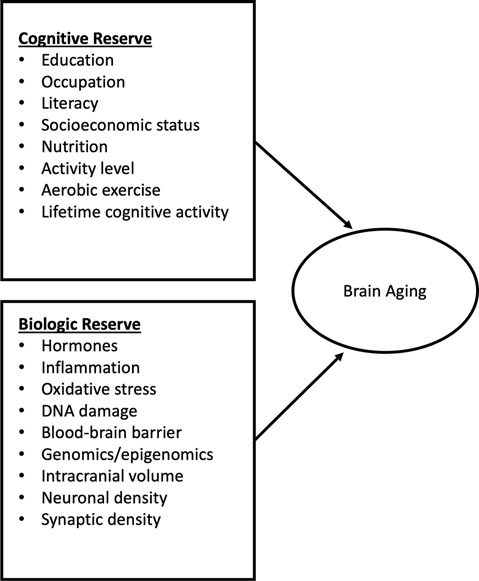
Influences of cognitive and brain reserve on brain aging.

Along with experiencing normal biological changes associated with aging, including changes to the brain, women treated for breast cancer may also experience cancer and cancer treatment-related cognitive decline. One theory of accelerated aging is based on the idea that aging is the result of reactive oxygen species production (ROS) and mitochondrial stress giving rise to DNA damage, and that in the tumor environment cancer cells can stimulate ROS production in adjacent normal cells resulting in inflammation and the metabolism of cancer (Lisanti et al., 2011). Accelerated aging precipitates inflammation, DNA damage, autophagy, and aerobic glycolysis that stimulates tumor growth and metastasis (Lisanti et al., 2011). The mechanism is that cancer cells produce ROS, which activates the innate immune system *via* nuclear factor kappa beta production and cancer metabolism through hypoxia-inducible factor 1 activation (Lisanti et al., 2011). To counteract the effects of aging, both cognitive and brain (biologic) reserves may be necessary (Figure 2). Cognitive and brain reserve may account for the preservation of CF in the presence of disease and treatment (Barulli and Stern, 2013). Yet, the effects of cancer and cancer treatment may diminish cognitive and brain reserves, thereby leading to a weakened defense against aging-related outcomes that include decreased CF (Mandelblatt et al., 2014; Bender et al., 2018). This theory is further complicated when considering older patients at the same chronological age, with the same diagnosis, who vary from being biologically younger than their actual age (elevated reserve) to being biologically older than their age (i.e., in poorer health; reduced reserve; Mandelblatt et al., 2014; Kresovich et al., 2019). Multiple environmental factors may enhance cognitive reserve including education, lifestyle (e.g., physical activity), and occupational history (Treanor et al., 2016). Promoting factors such as physical activity may enhance cognitive and brain reserve and improve CF (Cheng, 2016), although these associations have not been well-documented in women with breast cancer (Zimmer et al., 2016).

Changes in cognition have been detected using self-report, standardized neuropsychological instruments and neuroimaging approaches. Cancer-related changes in CF can be detected across several cognitive domains including difficulties in learning, concentrating, remembering, and decision-making. They may also experience deficits in executive functioning, which is an umbrella term referring to many higher-order functions including planning, coordination, attentional control, and working memory (Nelson et al., 2007; Treanor et al., 2016). These cognitive differences have been supported by neuroimaging studies. Morphological brain changes and reduced activity in several areas (prefrontal/frontal cortex, hippocampus, parahippocampus) have been found in cancer patients (Gehring et al., 2012; Scherling and Smith, 2013; Simó et al., 2013; Treanor et al., 2016; Chen et al., 2018a,b, 2019).

Individuals diagnosed with cancer have poorer CF than their healthy age-matched counterparts (Ahles et al., 2010; Wefel et al., 2010). Cancer patients and survivors report decreased CF with breast cancer and its treatment that includes interference with psychological well-being, decision making, ability to efficiently perform daily activities, as well as adherence to life-prolonging cancer therapy (Ahles and Root, 2018). There is also evidence suggesting that adjuvant therapy is associated with decreased CF (Wefel et al., 2004a; Ahles et al., 2008; Bender et al., 2015, 2018). A subgroup of cancer patients and survivors can experience a delay in cognitive decline months or even years after the completion of chemotherapy (Wefel et al., 2010; Ahles and Root, 2018). Adults who were treated for childhood cancer suffer from various age-related diseases that are normally experienced by older individuals, including neurocognitive dysfunction (Hudson et al., 2013; Hodes et al., 2016). Poorer cognitive performance has also been found in cancer patients before definitive diagnosis, surgery, or chemotherapy treatment (Ahles and Root, 2018). A recent review exploring the cognitive effects of cancer and cancer treatment offers a summary of the topic, arguing that cognitive decline is not a problem of pharmacological toxicity but rather the result of a multitude of factors including cancer biology, cancer treatment, as well as predisposing and modifiable risk factors (Ahles and Root, 2018).

Studies are also being conducted to detect if differences exist in cognitive dysfunction for women treated for breast cancer based on a treatment regime. Healthy controls (women without breast cancer matched for important variables related to CF, i.e., age, education) perform better on cognitive tasks compared to women with breast cancer post-surgery, and there is evidence for cognitive decline after exposure to adjuvant therapy with aromatase inhibitors (Bender et al., 2015). Another study found equivalent levels of cognitive impairment based on neuropsychological performance across treatment groups (radiation alone, chemotherapy alone, radiation and chemotherapy) in women with breast cancer before initiation of adjuvant endocrine therapy (Van Dyk et al., 2018). Interestingly, this is contrary to results that examined self-report of CF in the same study in which women who received a combination of chemotherapy and radiation reported significantly higher levels of cognitive problems (Ganz et al., 2013). This discordance between scores on neuropsychological and self-report measures is common and raises a question as to whether or not neuropsychological methods accurately represent the cognitive effects of cancer and its treatment (Janelsins et al., 2017; Van Dyk et al., 2018) or that they are assessing different aspects of behavior. For example, scores on self-report cognitive measures are more likely to be correlated with other symptoms frequently experienced by women with breast cancer such as fatigue or depressive symptoms (Pendergrass et al., 2018). Ahles found that women with breast cancer who had a greater pre-therapy cognitive reserve, assessed with the Wide Range Achievement Test Reading score, had better processing speed post-chemotherapy (Ahles et al., 2010), It is also important to note that the treatment received by breast cancer patients is based on cancer biology and we cannot rule out that the differences in CF are related to differences in cancer biology.

Further studies have aimed to explore alterations in brain activity that occur as a result of chemotherapy in older women diagnosed with breast cancer. In a series of studies, magnetic resonance imaging was used to explore changes that occur in the brain using different chemotherapy regimens. It was found that gray matter density was decreased in women over the age of 60 with breast cancer who had been exposed to chemotherapy (Chen et al., 2018b) and that women who received certain chemotherapy drugs (docetaxel and cyclophosphamide) experienced a reduction of volume in their temporal lobe that was not present before chemotherapy (Chen et al., 2019). Alterations in intrinsic brain activity have also been detected in areas such as the bilateral subcallosal gyri, right anterior cingulate cortex and left precuneus in older women with breast cancer treated with endocrine therapy (Chen et al., 2019).

## Neuroplasticity and Epigenetics

Due to the dearth of information related to neuroplasticity specifically concerning epigenetic processes linked to CF and higher-order brain function in women with breast cancer, this review offers an examination of epigenetic modifications related to synaptic plasticity in various other conditions. Synaptic plasticity is a fundamental neuronal property that is the basis for memory formation in the brain, and several genes required for the formation of memory are regulated by epigenetic modifications (Sen, 2015). Examples of conditions were epigenetic modifications are associated with potential cognitive failures include Alzheimer’s disease, schizophrenia, and stress.

In Alzheimer’s disease, epigenetic alterations include noncoding RNAs (ncRNA), DNA methylation, and histone modifications. These modifications result in expression changes in genes such as brain-derived neurotrophic factor (*BDNF*) and cAMP response element-binding protein (CREB), both important for synaptic processes such as long-term potentiation and memory (Li et al., 2018). Changes in each of these processes contribute to Alzheimer’s disease. Epigenetic changes related to decreased function of N-methyl-D-aspartate receptor (NMDAR), a glutamate receptor that is essential for synaptic plasticity, learning and memory have been suggested to contribute to synaptic dysfunction and symptoms in schizophrenia (Snyder and Gao, 2019) as well as DNA methylation of certain polymorphisms in the BDNF gene (Ursini et al., 2016). There are also epigenetic factors associated with neural plasticity that results from chronic stress that includes gene expression changes resulting in the activation of the excitatory neurotransmitter glutamate, which increases depolarization of neurons (Tarai et al., 2019).

Research exploring epigenetic modifications linked to synaptic plasticity are continually ongoing in each of the above-mentioned conditions. As the body of literature related to these specific subjects increases, so will the knowledge in the general area of neuroplasticity and epigenetics. The results of these studies can be used to inform the area of CF and higher-order brain functions in other conditions such as in women with breast cancer.

## Epigenomics of Cognitive Function

Epigenomics is a branch of science that considers those modifications to the DNA that influence gene expression but do not alter the underlying DNA sequence (Baumgartel et al., 2011). The prefix “epi” implies “above,” therefore it can be thought that epigenomics involves all those modifications that take place above the genetic code (McCue and McCoy, 2017). Epigenetic modifications are the result of epigenetic markers that modify gene regulation, which in turn has an effect on transcription and protein production and hence the function of the cell. These markers include histone modification impacting chromatin condensation, noncoding RNA, and DNA methylation (Fessele and Wright, 2018).

DNA methylation is a key regulator of neuronal activation, neuronal plasticity, and memory formation (Levenson et al., 2006; Miller and Sweatt, 2007; Lubin et al., 2008; Miller et al., 2008, 2010; Guo et al., 2011; Grigorenko et al., 2016). The adult brain possesses the ability to dynamically alter its DNA methylation patterns. These changes, in turn, have an impact on neuronal functioning, learning, new memory formation, and other cognitive processes (Zovkic et al., 2013; Fischer, 2014; Guan et al., 2015). Mutations that affect DNA methylation can cause cognitive abnormalities including intellectual disabilities and Alzheimer’s disease (Amir et al., 1999, 2000; Xu et al., 1999; Fuso et al., 2011a,b; Jiraanont et al., 2017; Hartin et al., 2018; Polonis et al., 2018). Impairments in cognition in children are associated with DNA methylation linked to malnutrition (Peter et al., 2016). DNA methylation patterns generated from blood samples have also been significantly correlated with neuroimaging outcomes, for example, the relationship between BDNF promotor methylation and cortical thickness (Na et al., 2016).

There is a need for increased research in the area of epigenetics of CF of women diagnosed with breast cancer. In a review of the literature of clinical studies, only three studies were found that used epigenetics to investigate changes in cognition (Table 2, Bradburn et al., 2018; Liu et al., 2018; Yao et al., 2019). All three of these studies explored the relationship of epigenetics to inflammation and the effect of inflammation on CF (Bradburn et al., 2018; Liu et al., 2018; Yao et al., 2019), with one of these studies using a population of breast cancer patients (Yao et al., 2019).

**Table 2:** Publications highlighting epigenomics, cognitive function, and exercise.

Name	Population	Phenotypic focus	Study description	Findings
Bradburn et al. (2018)	Physically and mentally healthy adults, young and old, from the MyoAge cohort (*n* = 361)	Cognition	Investigated a panel of 35 cytokines in participants to identify age-related immune markers associated with specific cognition measures.	In blood samples, there is age-related hypomethylation at specific CpG sites in the promoter region of the *CXCL10* gene. A polymorphism in the *CXCL10* gene (rs56061981) alters methylation at one of these CpG sites and is associated with working memory. The cytokine CXCL10 was significantly associated with special working memory in older adults.
Gale et al. (2018)	Members of the Lothian Birth Cohort all aged 79 (*n* = 248)	Cognition and exercise	Investigated the cross-sectional relationship between biological age (using DNA methylation for extrinsic and intrinsic epigenetic age acceleration) and sedentary and walking behavior in older adults.	No convincing evidence that biological age is associated with sedentary or walking behavior.
Liu et al. (2018)	African Americans from the Genetic Epidemiology Network of Arteriopathy (GENOA) study (*n* = 289)	Cognition	Investigated the association between peripheral blood leukocyte methylation levels in the *APOE* genomic region (*APOE, TOMM40, PVRL2, APOC1*) and cognitive function.	Methylation levels at many of the CpGs in the APOE genomic region have an inverse association with delayed recall during the normal cognitive aging process.
Marioni et al. (2015)	Members of the Lothian Birth Cohort of 1936 [at ages 70 (*n* = 920); 73 (*n* = 299); 769 (*n* = 273)]	Cognition and exercise	Examined the association between the epigenetic clock and lung function, walking speed, grip strength, and cognitive ability	Cross-sectional correlations were significant between age acceleration and cognition as well as lung function and grip strength.
Yao et al. (2019)	Breast cancer patients and healthy non-cancer controls from the National Cancer Institute Community Oncology Research Program (*n* = 93)	Cognition	Characterization of changes in leukocyte DA methylome and examination of significant methylation changes with perceived cognitive impairments.	Chemotherapy alters the DNA methylation pattern in leukocytes of breast cancer patients and the CpG cg16936953 in the *VMP1/MIR21* gene is associated with cognitive decline in breast cancer patients.
McCullough et al. (2017)	Women with breast cancer that were part of the Long Island Breast Cancer Study Project (total *n* = 1,254; *n* = 807 with tumor methylation data)	Exercise	Examined modification of recreational physical activity-mortality association by gene-specific promoter methylation and global methylation.	Promotor methylation of *TWIST1, HIN1, CCND2, APC* might alter the inverse association between recreational physical activity and mortality after breast cancer diagnosis. Higher methylation/lower mortality. No interaction between recreational physical activity and global methylation.
McEwen et al. (2018)	Community-dwelling older women aged 55–70 from Vancouver, Canada (*n* = 20)	Exercise	Investigated epigenetic modifications after 6-month self-management intervention with group education, individual personal training sessions, and use of an activity monitor (Fitbit).	No significant association between DNA methylation and physical activity but did find epigenetic changes in weight-associated genes *RUNX3* and *NAMPT*.

In healthy young adults compared to healthy older adults hypomethylation at specific CpGs within the specific inflammatory chemokine (CXCL10) corresponded with higher expression of the *CXCL10* gene in blood leukocytes and was negatively associated with working memory function. Using fresh frozen human samples of the prefrontal cortex, the same researchers also found higher levels of the CXCL10 protein in individuals with Alzheimer’s disease compared to older healthy adults (Bradburn et al., 2018). Overall, they showed that age-related loss of DNA methylation of the *CXCL10* promoter was associated with an upregulation of plasma cytokine. Another study explored the influence of chemotherapy on the DNA methylome of leukocytes in women with breast cancer compared to healthy controls and whether these changes were associated with decreases in perceived CF (Yao et al., 2019). The results of this study showed that the DNA methylome of breast cancer patients was altered after chemotherapy treatment when compared to the stable methylome of non-treated controls. It also showed that there were correlations between methylation changes and CF suggesting that blood methylation could be used as a non-invasive biomarker for prediction of symptom development and treatment response (Yao et al., 2019).

The role of DNA methylation of the genomic region of apolipoprotein E (APOE) and its association to CF in individuals without dementia was studied in older African Americans from the Genetic Epidemiology Network of Arteriopathy. The results of this study suggest that epigenetic mechanisms play an important role in influencing CF. Researchers found eight CpG islands in three different genes, APOE and two proximal genes (*PVRL2* and *TOMM40*), that show an inverse relationship between methylation level and memory, and in particular with delayed recall (Liu et al., 2018). The results from this study suggest that changes in methylation may serve as an early biomarker for diseases that affect CF, such as dementia or maybe an intervention target for symptom amelioration (Liu et al., 2018).

In sum, early evidence in this field suggests that changes in markers of DNA methylation may explain age-related cognitive losses as well as cognitive decline associated with Alzheimer’s disease or breast cancer. The field is in desperate need of more research testing this hypothesis.

## Effects of Exercise on Cognitive Function

There is clear evidence that exercise positively influences several aspects of BH including CF. However, the strength and quantity of evidence in the field varies as a function of the age group and population with greater evidence supporting the benefits of exercise on CF in older adults compared to other age groups or populations. Nevertheless, there is promising evidence for the positive effect of exercise on CF in several patient groups including schizophrenia, multiple sclerosis, attention-deficit hyperactivity disorder, and mild cognitive impairment. Complicating the issue is that exercise does not influence all cognitive domains equally and some domains (i.e., executive function) might be influenced more by exercise than other domains. The reasons for this remain poorly understood, but it might suggest that populations that show greater deficits in some cognitive domains (e.g., executive function) might especially benefit from engaging in exercise.

Unfortunately, the effects of exercise on cognitive performance in cancer patients remains relatively poorly understood (Derry et al., 2015). A recent Cochrane review of randomized controlled trials exploring non-pharmacological interventions, such as exercise, for influencing CF related to cancer treatment found a need for more evidence on the effectiveness of these strategies (Treanor et al., 2016). Of the five studies included in the review, only one considered the effect of exercise (Campbell et al., 2018). The intervention included 24-weeks of 150 min per week of aerobic exercise and found no effect of the intervention after adjusting for baseline cognitive performance (Treanor et al., 2016).

A separate Cochrane review investigated the effects of exercise on women who receive either chemotherapy or radiation for breast cancer (Furmaniak et al., 2016). This review examined the effect of exercise on a variety of breast cancer treatment-related side effects, including cognitive dysfunction. The review found that most research in this area focused on rehabilitation and health promotion in women who have already finished cancer treatment. Of the 32 studies included in the review, only two focused on the effect of exercise on CF (Steindorf et al., 2014; Schmidt et al., 2015). Overall, the review suggested that exercise may slightly improve CF, but further research is necessary to determine the optimal parameters (i.e., type, intensity, and frequency) of an exercise intervention (Furmaniak et al., 2016). For example, in a study that compared HIIT to moderate-intensity continuous training, both interventions had positive effects on CF, but HIIT had larger positive effects on episodic memory, working memory, and executive function (Northey et al., 2019). This study highlights the need to carefully construct research designs that optimize the intensity, frequency, and other characteristics of the exercise as it might lead to different patterns on cognitive outcomes.

Relatedly, there remain many unanswered questions on the most appropriate model or type of activity that is most beneficial for influencing CF in women with breast cancer. For instance, a recent study found that yoga did not have an immediate positive effect on CF in cancer survivors, but at the 3-month follow-up, yoga participants had significantly lower self-reported cognitive impairments, and those survivors who practiced yoga more frequently had a reduction in cognitive complaints (Derry et al., 2015). Results of other studies suggest that gentle movement exercises, such as Qigong, may also improve CF and enhance the positive impact of exercise (Larkey et al., 2016; Myers et al., 2019).

As described above, there is evidence that breast cancer affects some cognitive domains more than others and that many of these same cognitive domains are positively affected by exercise. For example, several studies have found positive effects of exercise on measures of information processing (Marinac et al., 2015; Hartman et al., 2018; Salerno et al., 2019). In one study, improvements in information processing were found in a 12-week intervention that prescribed 150 min per week of moderate-to-vigorous physical activity in survivors who had been diagnosed with breast cancer within the prior 2 years (Hartman et al., 2018). However, there were no significant changes in the other domains of cognition (e.g., verbal learning), suggesting that greater than 12 weeks of exercise is needed for improvements in CF.

Importantly, it should be noted that the majority of the above studies were conducted in cancer survivors who had already completed cancer treatment. There remains a dearth of knowledge regarding exercise and CF in cancer patients that are currently undergoing treatment, or the effect of an exercise intervention begun before cancer treatment.

## Epigenomics of Exercise

A recent review of epigenetics and exercise cites histone hyperacetylation and DNA methylation as essential actions for a transcriptional increase of crucial metabolic, myogenic, and regulatory genes as an early response to exercise and the mediation of ensuing changes in skeletal muscle (McGee and Hargreaves, 2019). Evidence has linked the AMP-activated protein kinase (AMPK), mitogen-activated protein kinase (MAPK), protein kinase A (PKA), protein kinase C (PKC), and calcium/calmodulin protein kinase II (CAMKII) biological signaling pathways with specific post-transcriptional histone modifications to exercise-induced transcriptional responses (McGee and Hargreaves, 2019). Exercise also results in a decrease in overall global DNA methylation. Specific regulatory and metabolic genes [peroxisome proliferator-activated receptor-gamma coactivator-1α (*PGC-1α*), peroxisome proliferator-activated receptor δ (*PPAR-δ*), mitochondrial transcription factor A (*TFAM*), and myocyte enhancer factor 2 (*MEF2*)] experience DNA hypomethylation attributed to exercise, with concomitant increased levels of gene expression associated with exercise (Barrès et al., 2012; McGee and Hargreaves, 2019). This review article also provides evidence that maternal and paternal exercise-induced epigenetic changes can be passed to offspring, but the mechanism for this has yet to be elucidated (McGee and Hargreaves, 2019).

Exercise impacts DNA methylation as well as genes and pathways involved in the engagement of epigenomic regulation and machinery in the central nervous system (Feng et al., 2007; Chao and Zoghbi, 2009; Sweatt, 2009; Ntanasis-Stathopoulos et al., 2013; Horsburgh et al., 2015a; Voisin et al., 2015; Kashimoto et al., 2016; Fernandes et al., 2017). Considerable evidence suggests that DNA methylation of candidate genes are impacted by exercise, including BDNF (West et al., 2001; Martinowich et al., 2003; Bekinschtein et al., 2008a,b, 2014; Lu et al., 2008; Gomez-Pinilla et al., 2011; Ryan et al., 2019) and inflammation-related genes (Horsburgh et al., 2015b). Exercise may reverse DNA methylation changes that are induced by aging (Penner et al., 2010, 2011, 2016; Oliveira et al., 2012; Su and Tsai, 2012; Elsner et al., 2013; Barter and Foster, 2018; Harman and Martín, 2020). For example, in a recent study evaluating blood-based DNA methylation as part of a randomized controlled trial of an exercise intervention in women (*n* = 12) with breast cancer, 43 genes were differentially methylated between those randomized to exercise and those to usual care (Zeng et al., 2012).

There is a dearth of research investigating the effects of exercise on DNA methylation in older women diagnosed with breast cancer. A recent review of the literature found two studies that examined the effect of exercise on DNA methylation in older women (Table 2, McCullough et al., 2017; McEwen et al., 2018). In a study aimed at the underlying mechanism by which physical activity provides health benefits, researchers studied DNA methylation in a small sample of 20 healthy but previously inactive postmenopausal women before and after a lifestyle intervention and found no significant association between DNA methylation and physical activity but did find epigenetic changes associated with percent body weight in peripheral blood samples (McEwen et al., 2018). The lack of an epigenetic finding could be the result of the small sample size or the fact that physical activity was measured *via* daily step count and did not discuss an increase in exercise intensity. Another study examined the association between recreational physical activity before breast cancer diagnosis and breast cancer survival *via* promotor regulation in cancer-related genes (McCullough et al., 2017). This study discovered that promotor methylation of breast cancer-related genes (*HIN1, TWIST1, APC, and CCND2*) could modify the inverse association between prediagnostic physical activity and mortality post breast cancer diagnosis but power in this study was limited and further research is necessary to verify these findings (McCullough et al., 2017).

There is limited research using DNA methylation to examine the effects of exercise on cognition. Using a cohort of participants with a mean age of 70 years in a study designed to examine cognitive aging, researchers investigated the relationship between epigenetic age (using DNA methylation; Hannum et al., 2013; Horvath, 2013; Horvath and Raj, 2018) and level of physical activity in older adults. Results did not show convincing evidence that epigenetic age was associated with physical activity (Gale et al., 2018). A major limitation of this study was that everyday activity was considered physical activity rather than moderate-to-vigorous intensity exercise. Another study using the same cohort of participants investigated the association between age acceleration (the residuals from the regression of epigenetic age on chronological age), lung function, grip strength, walking speed and CF found significant correlations between age acceleration and cognition where greater age acceleration correlated with poorer cognitive performance (Marioni et al., 2015).

## Potential Areas of Opportunity for Investigation

**Figure 3:**
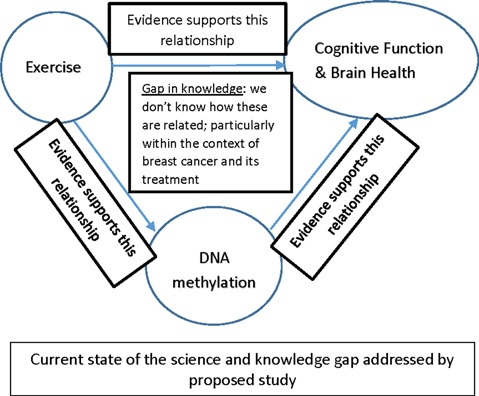
Gap in knowledge.

In examining the areas related to the effect of breast cancer and treatment on brain aging, CF, effects of exercise on CF, as well as the epigenetics of CF and exercise we found a dearth of research in the area of changes in CF in postmenopausal women diagnosed with breast cancer. Conceptually, this gap in knowledge is represented in Figure 3.

As shown in the figure and discussed in this review article, potential areas for future investigation include those studies designed to optimize the relationship between CF and BH, exercise and DNA methylation in cancer and cancer treatment, particularly within the context of breast cancer and breast cancer treatment. Research in these areas has the potential to increase our understanding of the molecular underpinnings of cancer-related phenotypes such as decreased cognition and can lead to more targeted treatment and prevention strategies to ameliorate or avoid cognitive decline associated with breast cancer and its treatment.

## Author Contributions

KE, CB, and YC conceptualized the project, conducted literature searches, and provided feedback on the manuscript. MW also conducted literature searches and wrote the majority of the manuscript. All authors gave their approval of the final version. The corresponding author attests that all listed authors meet authorship criteria and that no others meeting the criteria have been omitted.

## Conflict of Interest

The authors declare that the research was conducted in the absence of any commercial or financial relationships that could be construed as a potential conflict of interest.
